# Upregulation of SPOCK2 inhibits the invasion and migration of prostate cancer cells by regulating the MT1-MMP/MMP2 pathway

**DOI:** 10.7717/peerj.7163

**Published:** 2019-07-12

**Authors:** Gang Liu, Fang Ren, Yongsheng Song

**Affiliations:** 1 Department of Urology, Shengjing Hospital of China Medical University, Shenyang, Liaoning, China; 2 Department of Obstetrics and Gynecology, Shengjing Hospital of China Medical University, Shenyang, Liaoning, China

**Keywords:** SPOCK2, Prostate cancer, Migration, Invasion, MT1-MMP, MMP2

## Abstract

**Background:**

It is known that secreted protein acidic and cysteine rich (osteonectin), cwcv and kazal-like domains proteoglycan 2 (SPOCK2) plays a significant role in the development and progression of several human cancers; however, the role of SPOCK2 in prostate cancer (PCa) remains unclear. This study aimed to find the role and mechanism of SPOCK2 in the development and progression of PCa.

**Methods:**

The messenger ribonucleic acid (mRNA) expression of *SPOCK2* in PCa tissue was detected by real-time polymerase chain reaction (PCR). Upregulation of the *SPOCK2* gene was achieved using the DU145 and LNCaP cells by transfecting the cells with *SPOCK2* recombinant fragment. Cell invasion and migration ability were detected by transwell chamber and wound healing assay. The expression of membrane-type 1 matrix metalloproteinase (MT1-MMP) and matrix metalloproteinase 2 (MMP2) in the cells was detected by Western Blot and zymography gel assay.

**Results:**

The mRNA level of *SPOCK2* was significantly lower in the PCa tissue compared to benign prostate hyperplasia. Upregulation of *SPOCK2* inhibited cell invasion and migration in DU145 and LNCaP cells, inhibited the expression of MT1-MMP and MMP2 and, inhibited activation of MMP2 in DU145 and LNCaP cells.

**Conclusion:**

SPOCK2 is associated with the progression of PCa. Upregulation of SPOCK2 can inhibit PCa cell invasion and metastasis by decreasing MT1-MMP and MMP2 gene expression and decreasing MMP2 protein activation.

## Introduction

Prostate cancer (PCa) is the most common cancer diagnosed among men in developed countries, and it has become the most common malignant tumor of the urinary system and the fastest growing male malignancy in China (Zheng et al., 2016; Chen et al., 2015; Chen et al., 2016b). Though the use of prostate-specific antigen screening has improved the detection rate of PCa (Welch & Albertsen, 2009) and reduced the mortality risk of PCa, it did not improve all-cause mortality rates (Fenton et al., 2018; Schröder et al., 2012). The 5-year survival rate for most patients with local or regional PCa is 90%, while for men diagnosed with advanced PCa, the 5-year survival rate is only 30%, and metastasis and recurrence of PCa the leading causes of the death (Shah et al., 2016; Antonarakis et al., 2010). Studying the mechanism of invasion and metastasis of PCa is therefore of great significance for improving the prognosis of PCa.

The secreted protein acidic and cysteine rich gene in combination with osteonectin, cwcv and kazal like domains proteoglycan 2 (SPOCK2), also known as testincan-2, is a member of the SPOCK family. This gene encodes a protein that binds to glycosaminoglycans to form part of the extracellular matrix (ECM), which plays an important role in cell invasion and metastasis (https://www.ncbi.nlm.nih.gov/gene/9806). It was found that SPOCK2 protein is expressed in the ECM of alveolar cells and that polymorphism of this gene was correlated with human alveolar progression, and it was consequently suggested that *SPOCK2* might be a gene of interest in alveolar dysplasia (Hadchouel et al., 2011). Another study further found that oligonucleotide polymorphism of the *SPOCK2* gene was also related to the deletion of 16q chromosome in breast cancer (Nordgard et al., 2008), which was the first report to describe the correlation between the *SPOCK2* gene and cancer. In addition, Chung et al. (2008) found that abnormal methylation of the *SPOCK2* gene was closely associated with PCa, breast cancer, and colon cancer by screening differential methylation genes, and it was deduced that screening for epigenetic inactivation of *SPOCK2* could be used as a diagnostic marker for PCa (Verma, Patel & Verma, 2011). This study was the first to highlight the correlation between *SPOCK2* and PCa. In a study of glioma, it was found that *SPOCK1* and *SPOCK3* can inhibit the invasiveness of glioma cells by suppressing membrane-type 1 matrix metalloproteinase (MT1-MMP)-mediated matrix metalloproteinase 2 (MMP2) activation, and that high level of SPOCK2 expression could abrogate the inhibition of MMP2 by *SPOCK1* and *SPOCK3* and increase the invasiveness of glioma cells (Nakada et al., 2001, 2003). In contrast, in a study of PCa, *SPOCK1* promoted tumor growth and metastasis (Chen et al., 2016a), however, the role of the *SPOCK2* gene in PCa remains to be reported. The current study aimed to investigate the role and mechanism of action of *SPOCK2* in the development and progression of PCa.

## Materials and Methods

### Tissue sample collection

All specimens were obtained from patients hospitalized in the Department of Urology of Shengjing Hospital (Shenyang, China). Specimens were collected from 30 surgical patients with PCa and 20 surgical patients with benign prostate hyperplasia (BPH). Informed consent was obtained from all patients, and agreement was obtained from the Ethics Committee of China Medical University before collecting the samples (2018PS302K). None of the patients had other surgical, endocrine, immunological or metabolic diseases, or had taken any other hormonal medication in the three months prior to surgery.

### Cell culture and transfection

The DU145 and LNCaP cells were purchased from the Shanghai Institute of Cell Biology of the Chinese Academy of Sciences (Shanghai, China). The cells were cultured in RPMI-1640 medium supplemented with 10% fetal bovine serum (FBS), in a sterile incubator at 37 °C under 5% CO_2_.

The *SPOCK2* recombinant fragment and the adenoviral vector were purchased from Gene Pharma (Shanghai, China). The transfection was performed according to the manufacturer’s instructions. Mock transfected cells were used as negative controls; cells transfected with empty vector were used as vector controls. At 48 h after transfection, the supernatant was discarded and replaced with complete medium and the cells cultured for another 24 h at 37 °C under 5% CO_2_ for further study.

### Detection of *SPOCK2* mRNA expression in tissue and cells by real-time PCR

Total RNA was obtained from tissue and cells using TRIzol reagent (BioTeke, Beijing, China). All procedures were performed under RNAase free conditions according to the manufacturer’s instructions. After quantification of RNA, the expression of *SPOCK2* messenger ribonucleic acid (mRNA) was detected by real-time polymerase chain reaction (PCR). Primer information is shown in Table 1. The reaction conditions were as follows: incubation at 94 °C for 5 min, 40 cycles of incubation at 94 °C for 10 s, 60 °C for 20 s and 72 °C for 30 s. Data were analyzed using the 2^−ΔΔCT^ method. Each sample was assayed in triplicate.

**Table 1:** The primers of SPOCK2 for Real-time PCR.

Name	Sequence	Tm	Product length
SPOCK2 F	ATGGAGGACGAGCAATGGCT	61.8	93
SPOCK2 R	TGGGTCGGACGAGGGAAC	60.8	
β-actin F	CTTAGTTGCGTTACACCCTTTCTTG	62	156
β-actin R	CTGTCACCTTCACCGTTCCAGTTT	64.4	

### Detection of SPOCK2, MT1-MMP and MMP2 protein expression in cells by WB

Cells were harvested for 48 h after transfection. Radioimmunoprecipitation assay buffer was added to the samples and the samples placed on ice for 5 min. The samples were then centrifuged at 12,000 rpm, 4 °C for 10 min and the supernatant removed to obtain total protein. After total protein was quantified, sodium dodecyl sulfate (SDS)-polyacrylamide gel electrophoresis (PAGE) was carried out at 80V for 2.5 h. After being blocked for 1 h with 5% skim milk, the membranes were incubated with specific primary antibodies overnight at 4 °C followed by incubation with the secondary antibodies for 2 h at room temperature. The anti-SPOCK2 polyclonal antibody (1:200) was purchased from Santa Cruz, CA, USA, the anti-MT1-MMP (1:1,000) was purchased from Proteintech, Wuhan, China, and the anti-MMP2 (1:500) was purchased from Wanleibio, Shenyang, China. Secondary antibody (1:5,000) was purchased from Wanleibio, Shenyang, China. The film was scanned, and the optical density value of the target strip analyzed using a gel image processing system (Gel-Pro-Analyzer software). All assays were performed in triplicate.

### Detection of cell invasion by transwell chamber

Matrigel was thawed on ice and diluted with serum-free medium. The transwell chamber was coated with 40 μl pre-diluted Matrigel and placed in a 37 °C incubator for 2 h to solidify. The Matrigel-coated transwell chamber was placed in a 24-well plate and 800 μl of medium containing 30% FBS was added to the lower chamber while 200 μl of the cell suspension (2 × 10^4^) was added to the upper chamber. The 24-well plates were incubated at 37 °C under 5% CO_2_ for 26 h at which time cells could be seen in the lower chamber. Cells remaining on the top of the transwell chamber were scraped off with a cotton swab. Invasive cells were fixed with 4% paraformaldehyde for 20 min and stained with 0.5% crystal violet for 5 min; cells in the lower layer of the membrane were counted under an inverted microscope (200×).

### Detection of cell migration ability by wound healing assay

Cells were transfected in six-well plates and cultured until reaching 90% confluence. A wound was created with a pipette tip, the cells washed twice and cultured in serum-free medium. The wound was observed and photographed at 0 h and 24 h using an inverted microscope (Nikon, Tokyo, Japan). Cell migration ability was described as the number of cells that migrated into the wound. All assays were performed in triplicate.

### Detection of MMP2 expression in cells by zymography gel assay

The cell supernatant was obtained after centrifugation. After measuring the concentration, 60 μg protein was used for SDS-PAGE analysis with constant voltage overnight. The gel was then stained in the staining solution for 3 h. After being decolorized, the gel was photographed by a gel imaging system.

### Statistical analysis

All data are expressed as the mean ± standard deviation (SD) with SPSS version 13.0 software used for the statistical analyses. A Student’s *t*-test was used to determine the significance of two group differences. A one-way analysis of variance analysis was used for multiple comparisons. A *P*-value of *P* < 0.05 (two-tailed) was considered significant.

## Results

### *SPOCK2* mRNA expression in tissue

There was no significant difference between the age of the PCa group (69.27 ± 9.15) and the BPH group (66.97 ± 5.95) (*P* > 0.05). Real-time PCR showed a significantly lower expression of *SPOCK2* in the PCa tissues (2.25 ± 0.38) compared to the BPH tissues (6.12 ± 0.19) (*P* < 0.05).

### *SPOCK2* gene expression after transfection

**Figure 1:**
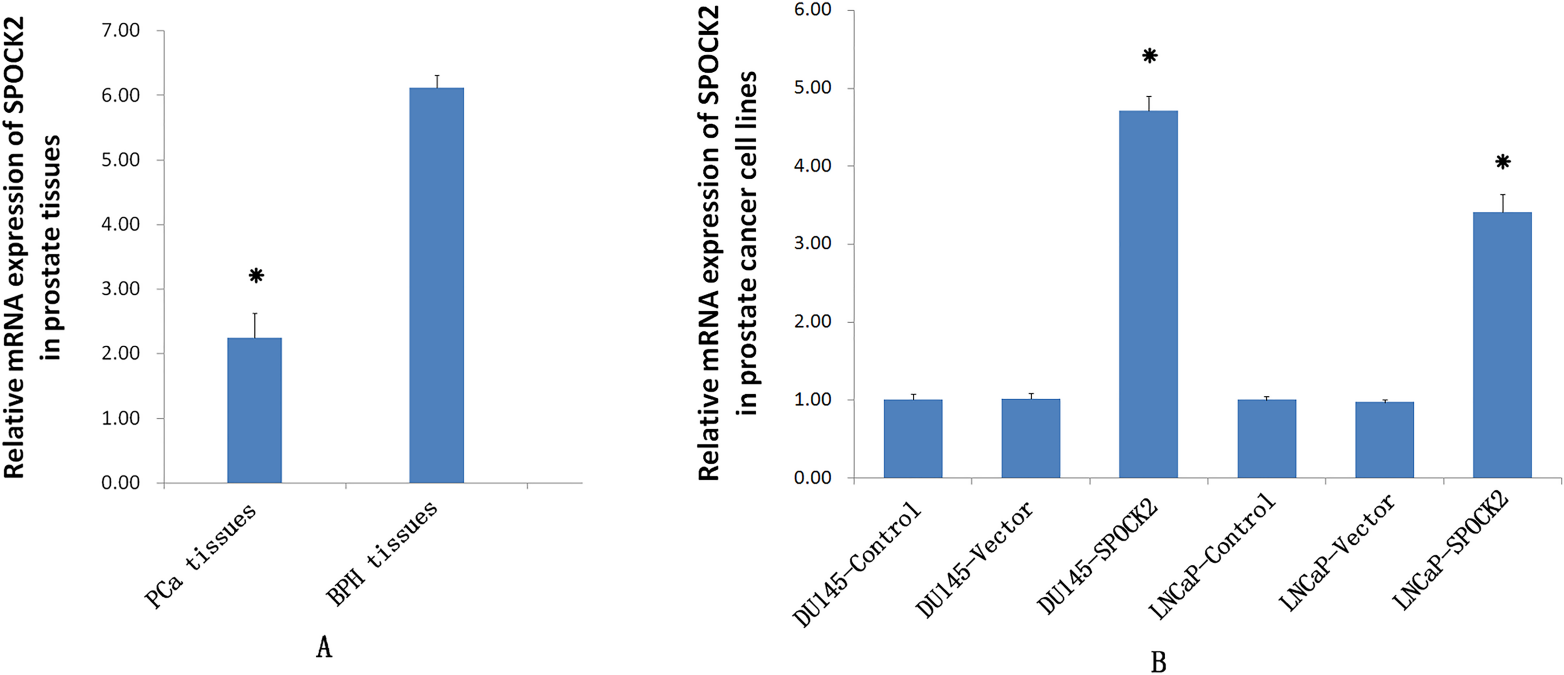
The *SPOCK2* mRNA expression was measured using RT-PCR. (A) The mRNA expression of *SPOCK2* in PCa tissues was significantly lower than that in BPH tissues (*P < 0.05). (B) The mRNA expression of *SPOCK2* in DU145 and LNCaP cells significantly increased after transfection (*P < 0.05).

**Figure 2:**
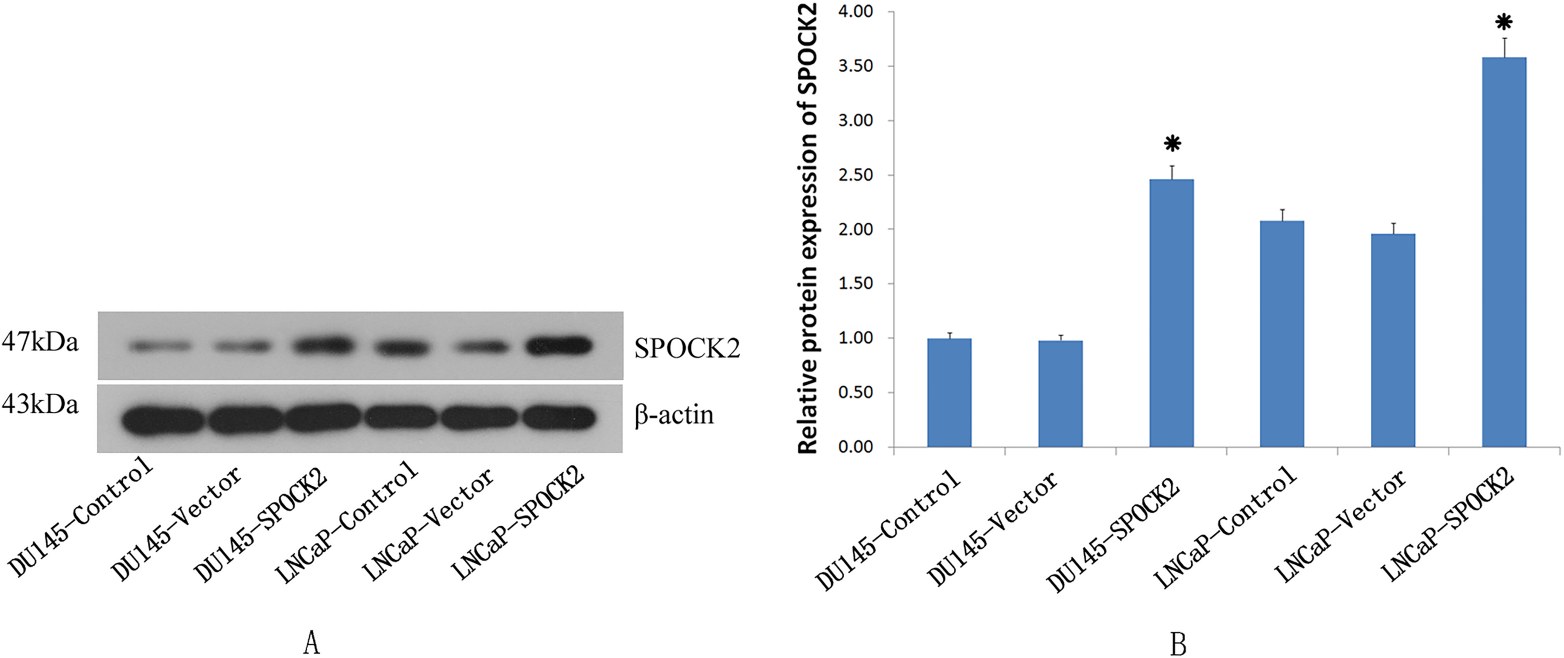
The SPOCK2 protein expression in cells. (A) The *SPOCK2* protein expression in DU145 and LNCaP cells was detected via WB. (B) The protein expression of *SPOCK2* in cells significantly increased after transfection (*P < 0.05).

The recombinant *SPOCK2* fragment was transfected into the DU145 and LNCaP cells by adenovirus, and the *SPOCK2* mRNA level and protein expression in the cells after transfection detected by real-time PCR (Fig. 1) and Western Blot (WB) (Fig. 2), respectively. The results showed that *SPOCK2* mRNA level and protein expression in DU145 and LNCaP cells after transfection were significantly increased (*P* < 0.05).

### Effect of *SPOCK2* upregulation on cell invasion

**Figure 3:**
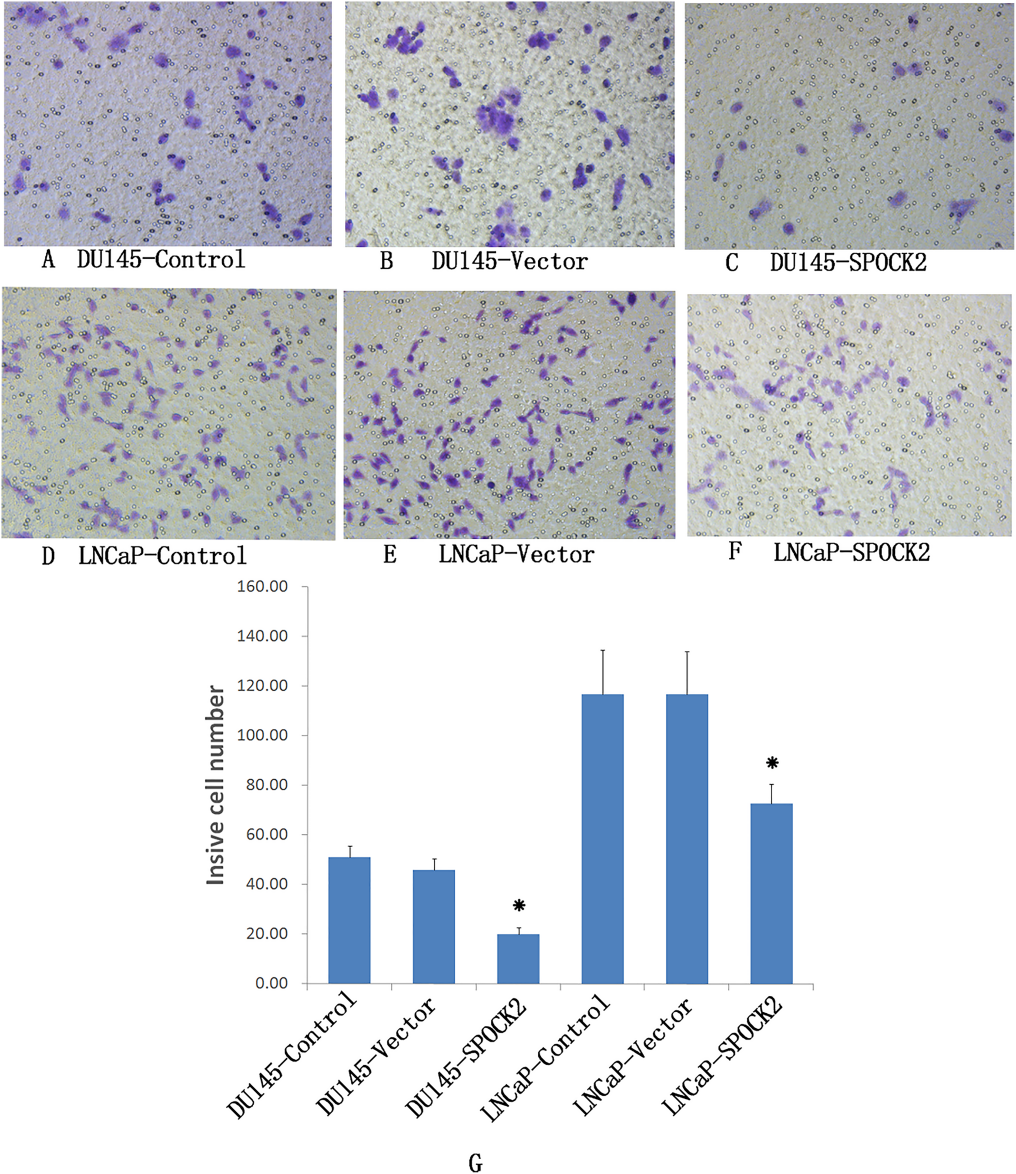
Upregulation of *SPOCK2* on cell invasive ability. (A–F) Upregulation of *SPOCK2* on cell invasive ability in DU145 and LNCaP cells following transfection with *SPOCK2* was detected by transwell invasion chamber. (G) The number of DU145 and LNCaP cells migrating into the lower chamber of the transwell invasion chamber was significantly reduced after transfection with *SPOCK2* (*P < 0.05).

Transwell invasion chamber was used to detect the invasion of DU145 and LNCaP cells after transfection (Fig. 3). The results showed that the invasive ability of the transfected group was significantly lower than that of the negative control group and the vector control group (*P* < 0.05).

### Effect of *SPOCK2* upregulation on cell migration

**Figure 4:**
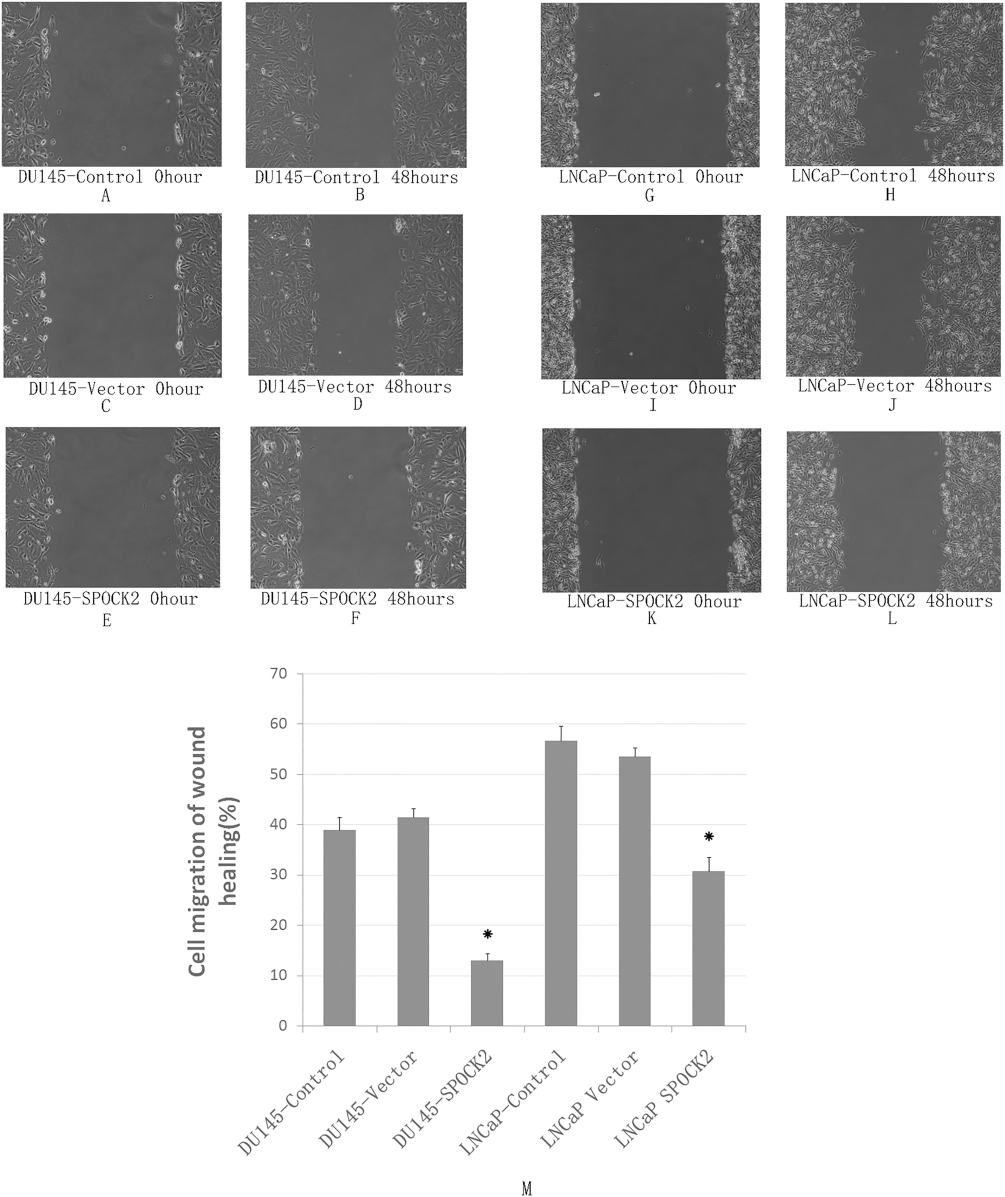
Upregulation of *SPOCK2* on cell migration. (A–L) Upregulation of *SPOCK2* on cell migration following transfection of DU145 and LNCaP cells with *SPOCK2* was detected by the wound healing assay. (B) (M) The wound healing assay showed that cell migration was significantly reduced after transfection with *SPOCK2* (*P < 0.05).

The wound healing assay was used to detect the migration ability of DU145 and LNCaP cells after transfection (Fig. 4). The results showed that the migration of both DU145 and LNCaP cells was significantly decreased after transfection compared with the negative control group and the vector control group (*P* < 0.05).

### Effect of *SPOCK2* upregulation on MT1-MMP2 and MMP2 expression

**Figure 5:**
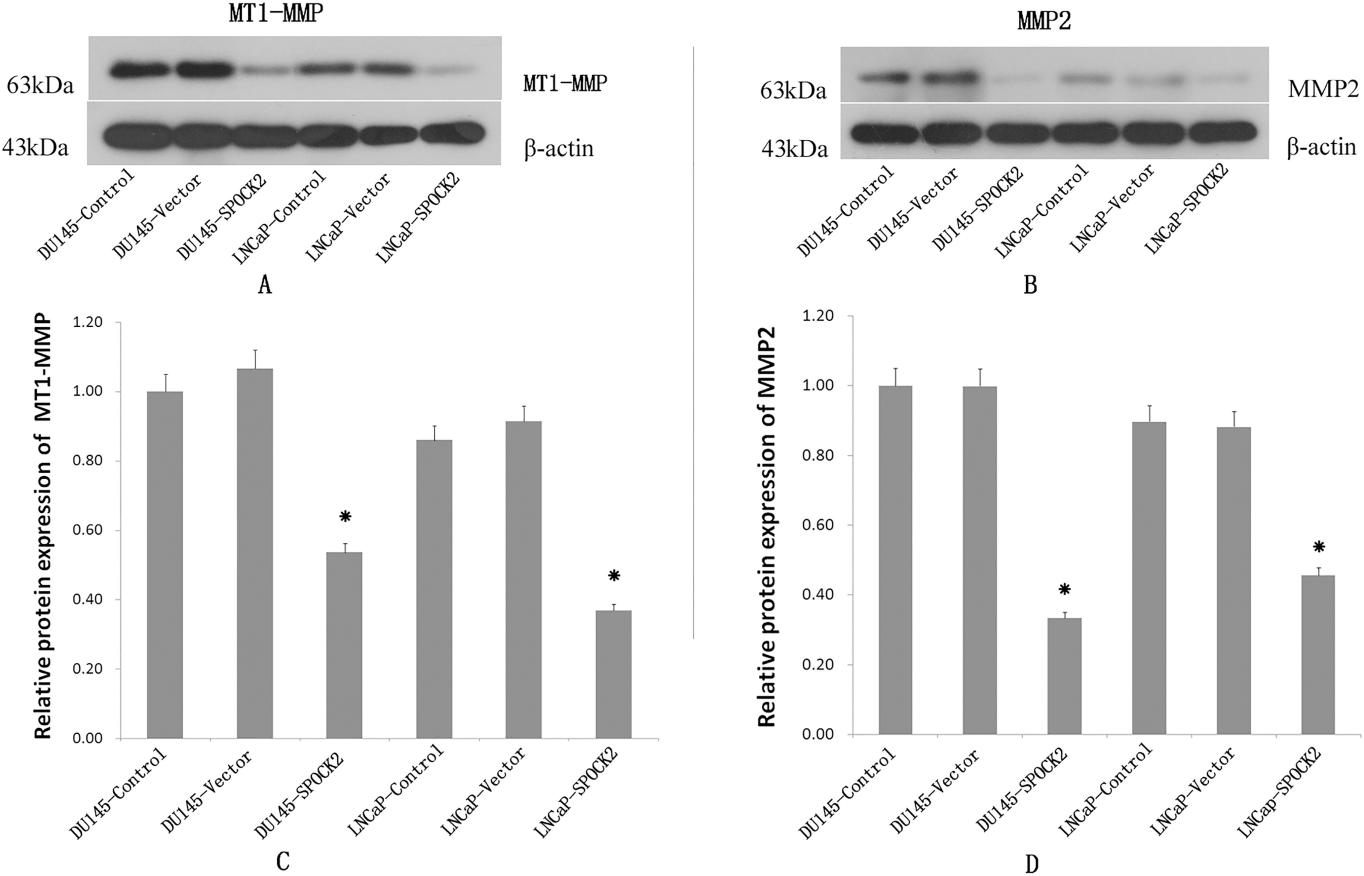
Upregulation of *SPOCK2* on the expression of MT1-MMP and MMP2. (A, C) Protein expression of MT1-MMP2 in DU145 and LNCaP cells transfected with *SPOCK2*, via WB, showing a significant decrease in MT1-MMP protein expression following transfection (*P < 0.05). (B, D) Expression of MMP2 in DU145 and LNCaP cells after transfection with *SPOCK2* was significantly decreased (*P < 0.05) as detected via WB.

The expression of MT1-MMP2 and MMP2 protein in DU145 and LNCaP cells was detected by WB (Fig. 5). The expression of MT1-MMP2 and MMP2 in cells in the transfected group were significantly lower than those in the negative control group and the vector control group (*P* < 0.05).

### Effect of *SPOCK2* upregulation on MMP2 activation

**Figure 6:**
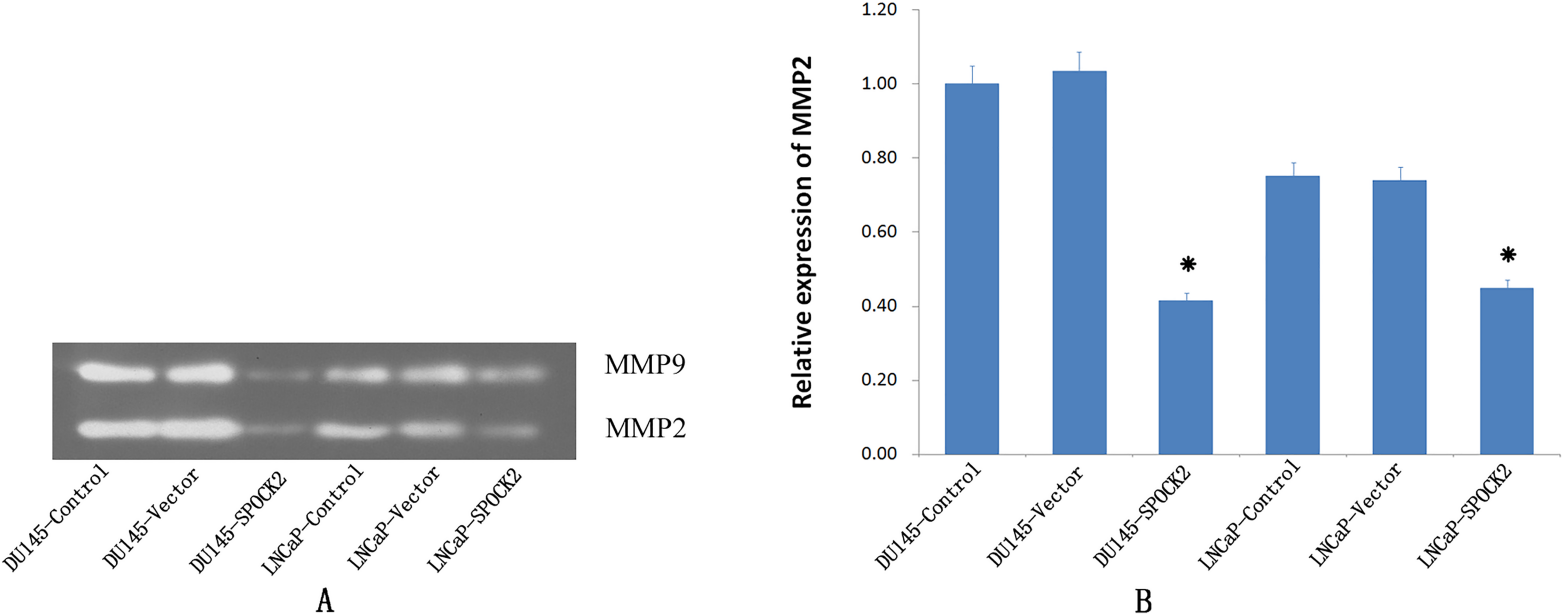
Upregulation of *SPOCK2* on the activaion of MMP2. (A) Upregulation of SPOCK2 on the activaion of MMP2 was detected by gelatin zymography. (B) Hostogram showed a significant decrease in MMP2 activation in DU145 and LNCaP cells following transfection of these cells with *SPOCK2* (*P < 0.05).

The expression of activated MMP2 was detected using gelatin zymography after transfection (Fig. 6). Expression of activated MMP2 protein after transfection was significantly lower than in the negative control group or the vector control group (*P* < 0.05).

## Discussion

The SPOCK family includes three members, SPOCK1, SPOCK2 and SPOCK3. In 2008, Chung et al. first reported the relationship between SPOCK2 and PCa, colon cancer and breast cancer, by gene screening. The relationship between colon cancer and *SPOCK2* was further verified by Sambuudash, Kim & Cho (2017), whereby it was found that the incidence of *SPOCK2* methylation in colon cancer was significantly higher than that in adjacent normal mucosa tissues. Another study (Ren, Wang & Li, 2011) reported that absence of SPOCK2 expression caused by hypermethylation of the *SPOCK2* gene, may be related to the development and progression of ovarian cancer. The relationship between PCa and the *SPOCK2* gene was not further investigated. In the current study, using real-time PCR, the level of expression of the *SPOCK2* gene was significantly lower in PCa tissues compared to that in BPH tissue, indicating that lower expression of the *SPOCK2* gene may be associated with the tumorigenesis and progression of PCa. To further clarify the role of SPOCK2 in PCa cells, PCa cells were transfected with an adenovirus vector to increase the expression of the *SPOCK2* gene. It was found that upregulation of this gene in PCa cells can inhibit cell invasion and migration. The results indicated the *SPOCK2* may play an important role in the progression of PCa by affecting the migration and invasion ability of the PCa cells. As *SPOCK2* is often inactivated by hypermethylation, a reversible epigenetic change (Przybilla et al., 2017), reversal of the expression of the SPOCK2 gene in PCa cells clinically, may improve the prognosis of patients with PCa.

The collagenase, MMP2, can decompose plasma fibronectin and laminin of the basement membrane, and is involved in various physiological and pathological processes in the human body (Song et al., 2015; Yi et al., 2014). The activation of MMP2 is regulated specifically by MT1-MMP, which itself is also capable of degrading a variety of ECM components (Koshikawa et al., 2000). Both MT1-MMP and MMP2 play an important role in the invasion and migration of PCa (Wang et al., 2009; Murray et al., 2012). To explore the mechanism by which SPOCK2 influences the invasion and migration of PCa cells, the expression of MT1-MMP and MMP2, and the activation of MMP2 in PCa cells was investigated using PCa cells with an upregulation of SPOCK2. The results indicated that upregulation of SPOCK2 in PCa cells could inhibit the expression of both MT1-MMP and MMP2 and decrease MMP2 activation. Together, the findings of the present study suggest that SPOCK2 may inhibit the invasion and migration of PCa cells by affecting MT1-MMP/MMP2 expression and activation. In a study of glioma, it was found that SPOCK1 and SPOCK3 could inhibit MT1-MMP-mediated MMP2 activation and inhibit glioma cell invasion by binding to MT1-MMP (Nakada et al., 2001). In contrast, high levels of SPOCK2 could abrogate this inhibition of MMP2 by SPOCK3 and increase the invasiveness of glioma cells (Nakada et al., 2003). In addition, in a study of gastric cancer, SPOCK1 was found to promote the invasion and metastasis of gastric cancer through Slug-induced epithelial-mesenchymal transition (Chen et al., 2018), and knockdown of SPOCK1 in colorectal cancer could inhibit proliferation and invasion of cancer cells by suppressing the PI3K/Akt Pathway (Zhao et al., 2016). These studies indicate that the effect of SPOCK2 on MT1-MMP/MMP2 in PCa cells may not work by directly binding to the MT1-MMP as the competitive inhibitor of SPOCK1 or SPOCK3. It is very possible that there may be other mechanisms by which SPOCK2 can affect the MT1-MMP/MMP pathway, which requires further investigation.

## Conclusions

In conclusion, the present study demonstrates that *SPOCK2* is associated with the progression of PCa, and that upregulation of *SPOCK2* can inhibit PCa cell invasion and metastasis. This is achieved ultimately by its ability to decrease *MT1-MMP* and *MMP2* gene expression and decrease activation of MMP2 protein.

## Supplemental Information

10.7717/peerj.7163/supp-1 Supplemental Information 1 Raw data for Real-time PCR. Raw data of Real-time PCR for data analyses and preparation for Fig. 1

10.7717/peerj.7163/supp-2 Supplemental Information 2 Raw data for Western Blot. Raw data of Western Blot for data analyses and preparation for Fig. 2 and Fig. 5

10.7717/peerj.7163/supp-3 Supplemental Information 3 Full-length uncropped blots for Fig. 2. The upper line: SPOCK2 expression for DU145 control, DU145 vector control, DU145 SPOCK2, LNCaP control, LNCaP vector, and LNCaP SPOCK2, respectively. The lower line: β-actin expression for DU145 Control, DU145 vector control, DU145 SPOCK2, LNCaP control, LNCaP vector, and LNCaP SPOCK2, respectively.

10.7717/peerj.7163/supp-4 Supplemental Information 4 Full-length uncropped blots for Fig. 5A. The upper line: MT1-MMP expression for DU145 control, DU145 vector control, DU145 SPOCK2, LNCaP control, LNCaP vector, and LNCaP SPOCK2, respectively. The lower line: β-actin expression for DU145 Control, DU145 vector control, DU145 SPOCK2, LNCaP control, LNCaP vector, and LNCaP SPOCK2, respectively.

10.7717/peerj.7163/supp-5 Supplemental Information 5 Full-length uncropped blots for Fig. 5B. The upper line: MMP2 expression for DU145 control, DU145 vector control, DU145 SPOCK2, LNCaP control, LNCaP vector, and LNCaP SPOCK2, respectively. The lower line: β-actin expression for DU145 Control, DU145 vector control, DU145 SPOCK2, LNCaP control, LNCaP vector, and LNCaP SPOCK2, respectively.

10.7717/peerj.7163/supp-6 Supplemental Information 6 Full-length uncropped blots for Fig. 6. The upper line: MMP2 expression in cells by zymography gel assay for DU145 control, DU145 vector control, DU145 SPOCK2, LNCaP control, LNCaP vector, and LNCaP SPOCK2, respectively. The lower line: MMP9 expression in cells by zymography gel assay for DU145 control, DU145 vector control, DU145 SPOCK2, LNCaP control, LNCaP vector, and LNCaP SPOCK2, respectively.

## Additional Information and Declarations

### Data Availability

The following information was supplied regarding data availability: All the raw measurements are available in the figures and in the Supplemental Files.
