# Supplementary material for: Upregulation of SPOCK2 inhibits the invasion and migration of prostate cancer cells by regulating the MT1-MMP/MMP2 pathway
Source: PeerJ. 2019 Jul 12;7:e7163. doi: 10.7717/peerj.7163 (PMC6628882; doi:10.7717/peerj.7163)
Supplement: Supplemental Information 1 — Raw data of Real-time PCR for data analyses and preparation for Fig. 1 [file peerj-07-7163-s001.docx]

Supplemental file 1. Raw data for Real-time PCR

| Sample | SPOCK2 | β-actin | △Ct |
| --- | --- | --- | --- |
| DU145-Control | 24.29 | 18.53 | 5.76 |
|  | 24.41 | 18.61 | 5.80 |
|  | 24.58 | 18.61 | 5.97 |
| DU145-Vector | 24.44 | 18.57 | 5.87 |
|  | 24.28 | 18.57 | 5.71 |
|  | 24.54 | 18.65 | 5.89 |
| DU145-SPOCK2 | 22.15 | 18.58 | 3.57 |
|  | 22.24 | 18.56 | 3.68 |
|  | 22.02 | 18.44 | 3.58 |
| LNCaP-Control | 28.44 | 16.11 | 12.33 |
|  | 28.55 | 16.20 | 12.35 |
|  | 28.49 | 16.26 | 12.23 |
| LNCaP-Vector | 28.54 | 16.22 | 12.32 |
|  | 28.61 | 16.30 | 12.31 |
|  | 28.55 | 16.13 | 12.42 |
| LNCaP-SPOCK2 | 26.72 | 16.10 | 10.62 |
|  | 26.58 | 16.15 | 10.43 |
|  | 26.60 | 16.04 | 10.56 |
