# Supplementary material for: Upregulation of SPOCK2 inhibits the invasion and migration of prostate cancer cells by regulating the MT1-MMP/MMP2 pathway
Source: PeerJ. 2019 Jul 12;7:e7163. doi: 10.7717/peerj.7163 (PMC6628882; doi:10.7717/peerj.7163)
Supplement: Supplemental Information 2 — Raw data of Western Blot for data analyses and preparation for Fig. 2 and Fig. 5 [file peerj-07-7163-s002.docx]

Supplemental file 2. Raw data for Western Blot

A. Western Blot for SPOCK2

| Sample | SPOCK2 | β-actin | Ratio |
| --- | --- | --- | --- |
| Du145-Control | 495.8 | 2720.9 | 0.18 |
| Du145-Vector | 496.53 | 2769.5 | 0.18 |
| Du145-SPOCK2 | 1212.1 | 2700.1 | 0.45 |
| Lncap-Control | 988.5 | 2603.3 | 0.38 |
| Lncap-Vector | 938.1 | 2622.4 | 0.36 |
| Lncap-SPOCK2 | 1737.9 | 2662.4 | 0.65 |

B. Western Blot for MT1-MMP

| Sample | MT1-MMP | β-actin | Ratio |
| --- | --- | --- | --- |
| Du145-Control | 1471.5 | 3031.2 | 0.49 |
| Du145-Vector | 1531.9 | 2959.8 | 0.52 |
| Du145-SPOCK2 | 766.82 | 2947.1 | 0.26 |
| Lncap-Control | 1223.1 | 2930.3 | 0.42 |
| Lncap-Vector | 1240.5 | 2795.5 | 0.44 |
| Lncap-SPOCK2 | 517.84 | 2892.2 | 0.18 |

C. Western Blot for MMP2

| Sample | MMP2 | β-actin | Ratio |
| --- | --- | --- | --- |
| Du145-Control | 639.54 | 2690.1 | 0.24 |
| Du145-Vector | 650.62 | 2738.9 | 0.24 |
| Du145-SPOCK2 | 213.42 | 2680.2 | 0.08 |
| Lncap-Control | 570.08 | 2670.7 | 0.21 |
| Lncap-Vector | 564.59 | 2690.9 | 0.21 |
| Lncap-SPOCK2 | 294.09 | 2709.7 | 0.11 |
