# Supplementary figures and images for: Upregulation of SPOCK2 inhibits the invasion and migration of prostate cancer cells by regulating the MT1-MMP/MMP2 pathway
Source: PeerJ. 2019 Jul 12;7:e7163. doi: 10.7717/peerj.7163 (PMC6628882; doi:10.7717/peerj.7163)

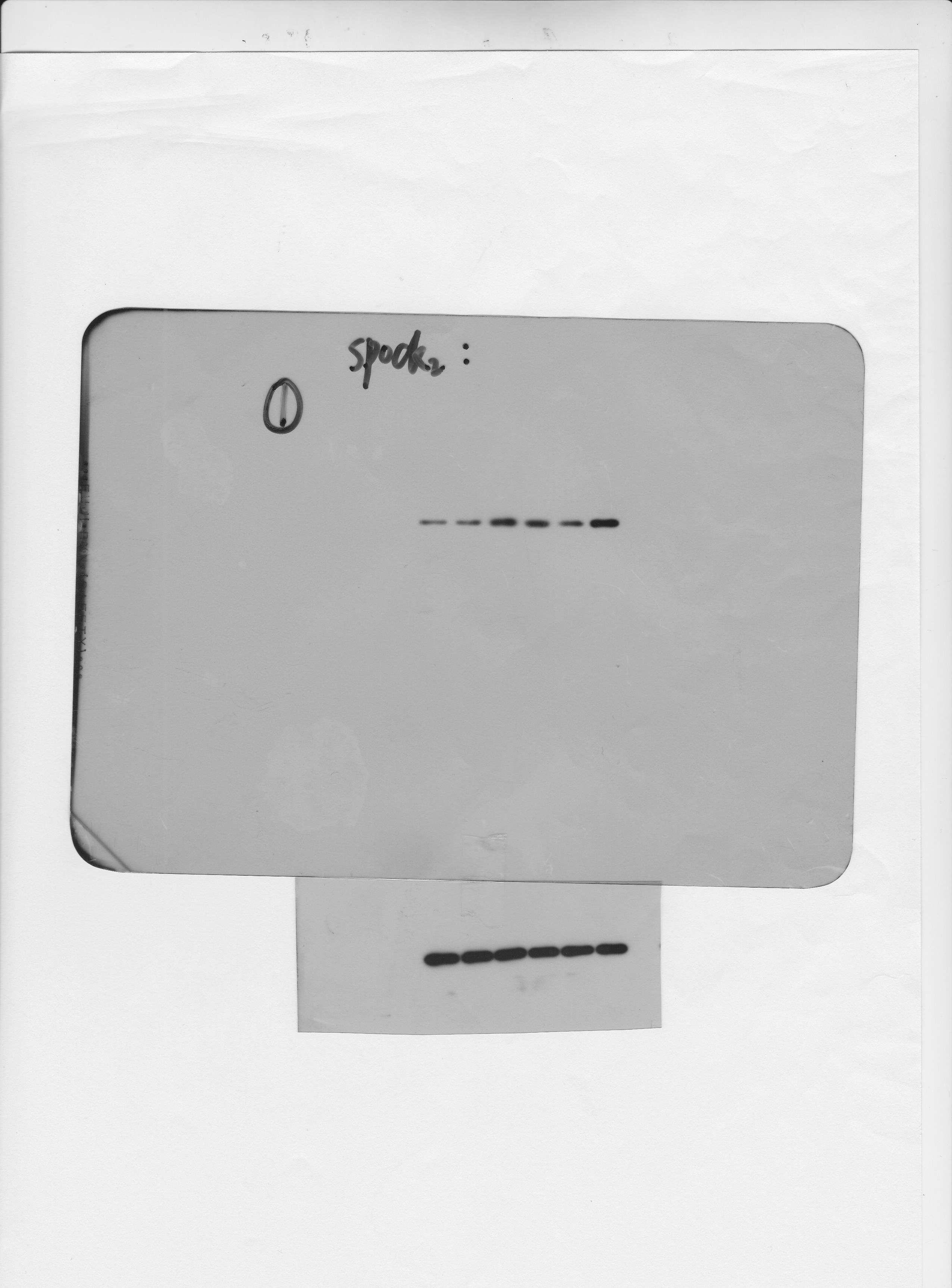

Supplement: Supplemental Information 3 — The upper line: SPOCK2 expression for DU145 control, DU145 vector control, DU145 SPOCK2, LNCaP control, LNCaP vector, and LNCaP SPOCK2, respectively. The lower line: β-actin expression for DU145 Control, DU145 vector control, DU145 SPOCK2, LNCaP control, LNCaP vector, and LNCaP SPOCK2, respectively. [file peerj-07-7163-s003.jpg]

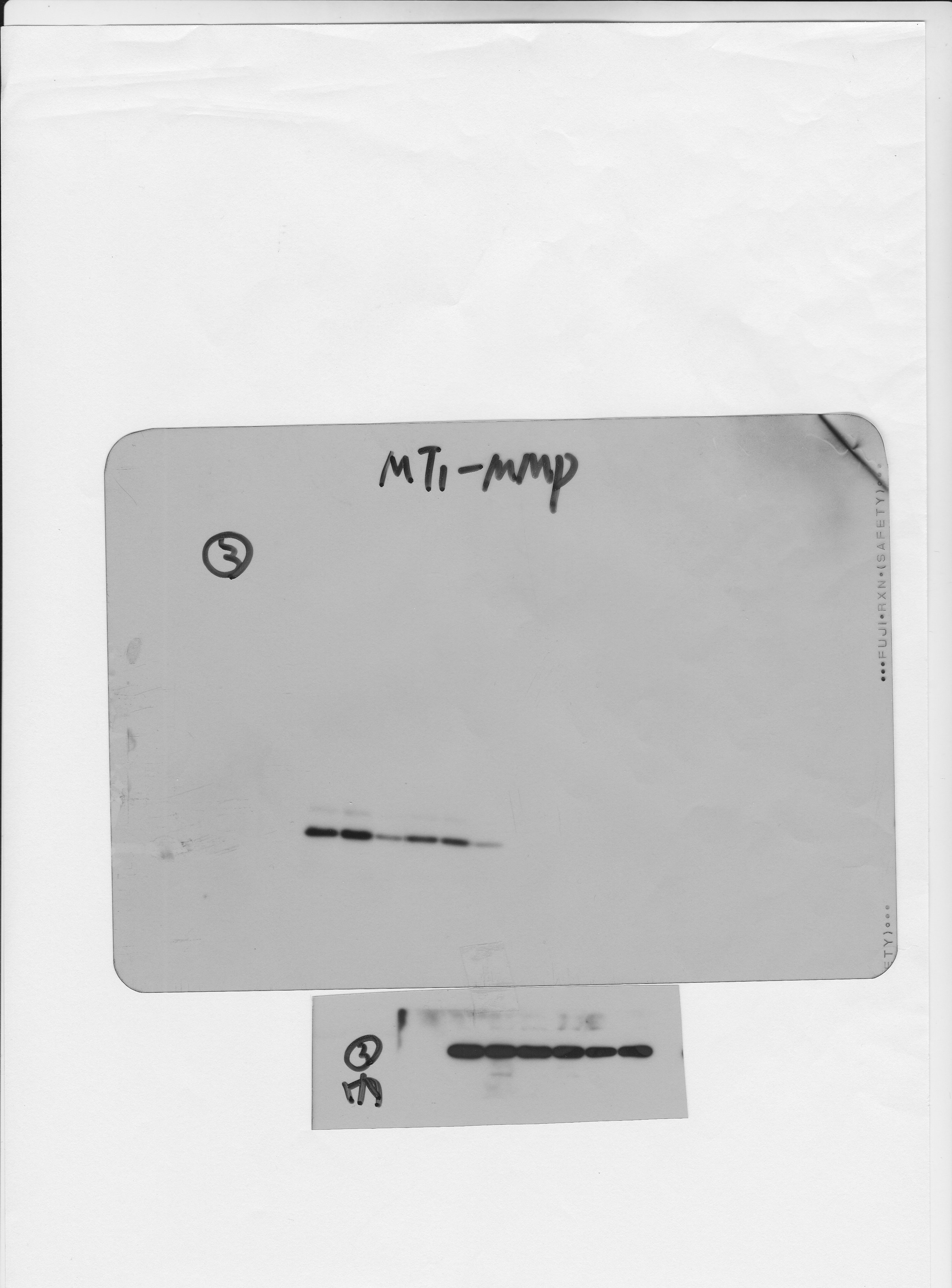

Supplement: Supplemental Information 4 — The upper line: MT1-MMP expression for DU145 control, DU145 vector control, DU145 SPOCK2, LNCaP control, LNCaP vector, and LNCaP SPOCK2, respectively. The lower line: β-actin expression for DU145 Control, DU145 vector control, DU145 SPOCK2, LNCaP control, LNCaP vector, and LNCaP SPOCK2, respectively. [file peerj-07-7163-s004.jpg]

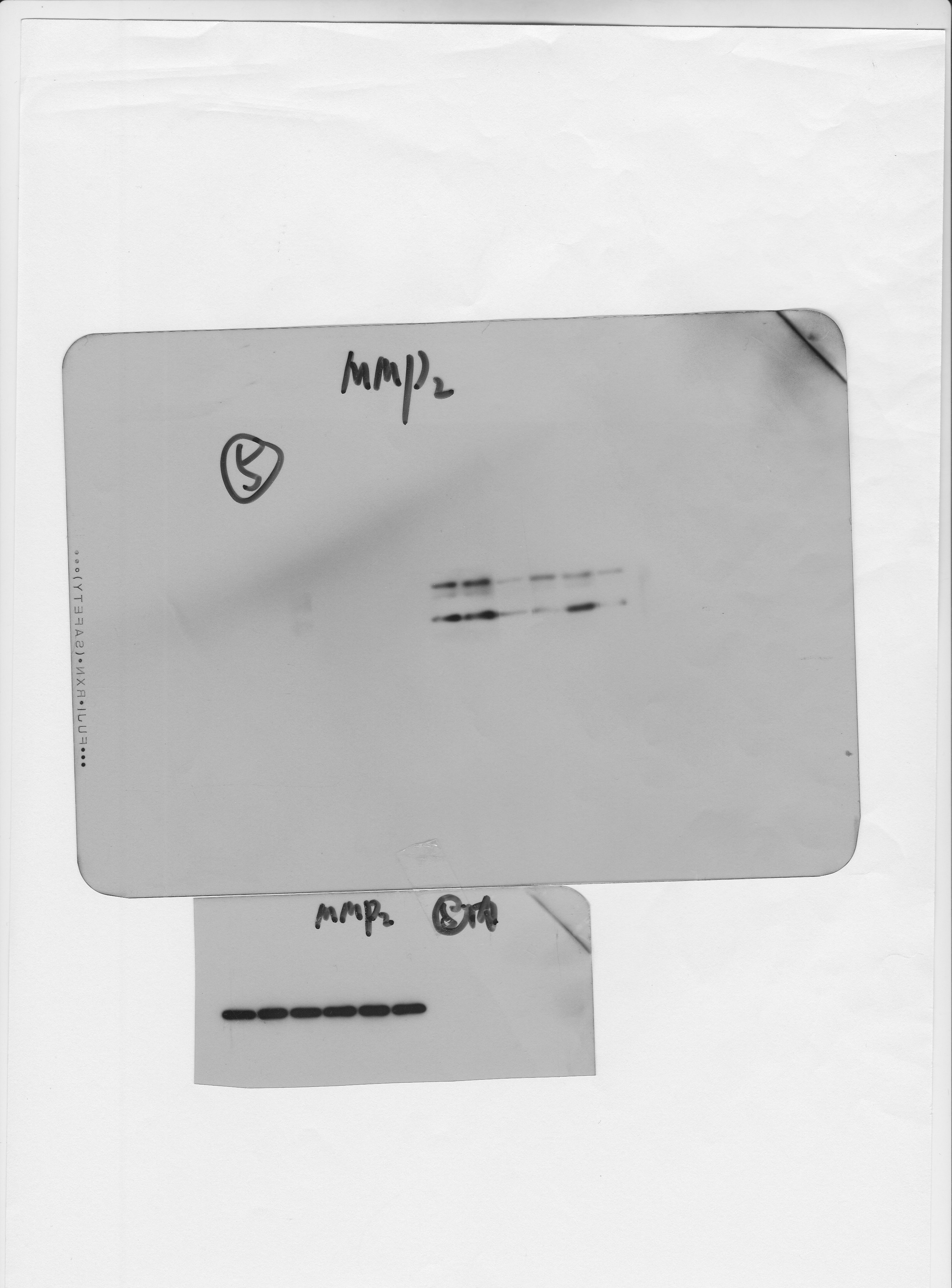

Supplement: Supplemental Information 5 — The upper line: MMP2 expression for DU145 control, DU145 vector control, DU145 SPOCK2, LNCaP control, LNCaP vector, and LNCaP SPOCK2, respectively. The lower line: β-actin expression for DU145 Control, DU145 vector control, DU145 SPOCK2, LNCaP control, LNCaP vector, and LNCaP SPOCK2, respectively. [file peerj-07-7163-s005.jpg]

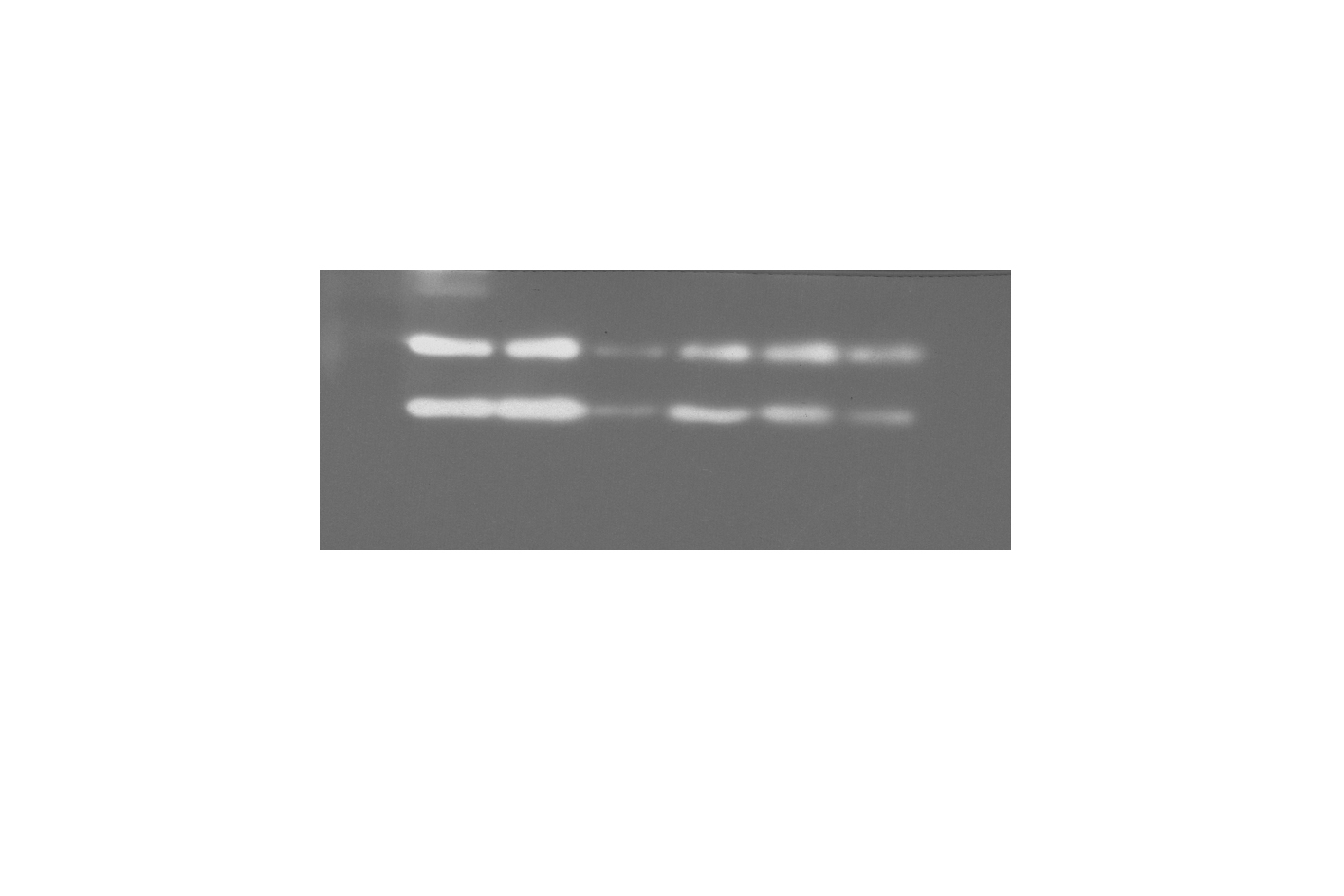

Supplement: Supplemental Information 6 — The upper line: MMP2 expression in cells by zymography gel assay for DU145 control, DU145 vector control, DU145 SPOCK2, LNCaP control, LNCaP vector, and LNCaP SPOCK2, respectively. The lower line: MMP9 expression in cells by zymography gel assay for DU145 control, DU145 vector control, DU145 SPOCK2, LNCaP control, LNCaP vector, and LNCaP SPOCK2, respectively. [file peerj-07-7163-s006.jpg]
